# Thermooptical evidence of carrier-stabilized ferroelectricity in ultrathin electrodeless films

**DOI:** 10.1038/s41598-018-26933-0

**Published:** 2018-05-31

**Authors:** O. Pacherova, D. Chvostova, T. Kocourek, M. Jelinek, A. Dejneka, E. Eliseev, A. Morozovska, M. Tyunina

**Affiliations:** 1 Institute of Physics of the Czech Academy of Sciences, Na Slovance 2, 18221 Prague, Czech Republic; 2 Institute of Problems for Material Sciences, NAS of Ukraine, 03028 Kyiv, Ukraine; 3 Institute of Physics, NAS of Ukraine, 03028 Kyiv, Ukraine; 4 Microelectronics Research Unit, Faculty of Information Technology and Electrical Engineering, University of Oulu, P. O. Box 4500, FI-90014 Oulu, Finland

## Abstract

Ferroelectric films may lose polarization as their thicknesses decrease to a few nanometers because of the depolarizing field that opposes the polarization therein. The depolarizing field is minimized when electrons or ions in the electrodes or the surface/interface layers screen the polarization charge or when peculiar domain configuration is formed. Here, we demonstrate ferroelectric phase transitions using thermooptical studies in ∼5-nm-thick epitaxial Pb_0.5_Sr_0.5_TiO_3_ films grown on different insulating substrates. By comparing theoretical modeling and experimental observations, we show that ferroelectricity is stabilized through redistribution of charge carriers (electrons or holes) inside ultrathin films. The related high-density of screening carriers is confined within a few-nanometers-thick layer in the vicinity of the insulator, thus resembling a two-dimensional carrier gas.

## Introduction

Growth technology, fundamental understanding, and applications of epitaxial perovskite oxide ferroelectric films has been progressing significantly in the last decades^1–6^. Such applications as transistor gates, tunnel-junction barriers, capacitor dielectrics, and others, employ films of a few nanometers in thickness, i.e., ultrathin films. The key scientific and practical issue is the minimum thickness at which ferroelectricity can be sustained in these films. If ferroelectric polarization in an ultrathin film on a crystal substrate is oriented perpendicular to the substrate surface (in the out-of-plane direction of the film), the associated bound polarization charge creates an internal electric field, opposing polarization. This depolarizing field may lead to instability of the polarization and disappearance of the ferroelectric phase below a critical thickness^7–9^. The most common mechanism against the depolarizing effect is screening of the polarization charge by electrons or ions in the top and bottom electrode layers, between which the film is sandwiched^10^. At the absence of one top electrode, an ionic compensation of the film’s surface or electronic reconstruction at films-substrate interfaces may occur, thereby allowing the ultrathin film to remain polar^11–15^. Generally, the film can also split into domains in response to the depolarizing field; therefore, it can sustain its polar state^16–24^. Interestingly, with decreasing thickness to tens of unit cells, bulk-like domains become unfavorable and peculiar domain configurations are formed^16–24^.

Here, by combining theoretical and experimental studies, we show that depolarization can be overcome by yet another mechanism, namely, self-screening through redistribution of charge carriers inside the films. To experimentally evidence ferroelectric state in electrodeless films, we investigate temperature evolution of optical index of refraction, *n*(*T*), or thermooptical behavior. First, using electrical characterization, we inspect ferroelectric phase transition in epitaxial 100-nm-thick Pb_0.5_Sr_0.5_TiO_3_ (PSTO) film, sandwiched between bottom SrRuO_3_ (SRO) and top Pt electrodes (Pt/PSTO/SRO). Then we analyze thermooptical behavior in similar, but electrodeless PSTO film. The comparison of electrical and thermooptical properties validates optical evidence of ferroelectricity in epitaxial thin films, in agreement with that in bulk perovskite oxide ferroelectrics^25–36^. Next, we investigate thermooptical behavior and demonstrate the ferroelectric phase transitions in electrodeless epitaxial PSTO films of ∼5 nm in thickness grown on insulating substrates. To analyze our observations, we perform theoretical calculations using the Landau-Ginzburg-Devonshire theory of ferroelectrics and considering the ferroelectric as a semiconductor. Our modeling and experimental results strongly suggest that ferroelectricity is stabilized owing to redistribution of the charge carriers in ultrathin films.

## Results and Discussion

### Electrical and thermooptical evidence of ferroelectricity

**Figure 1:**
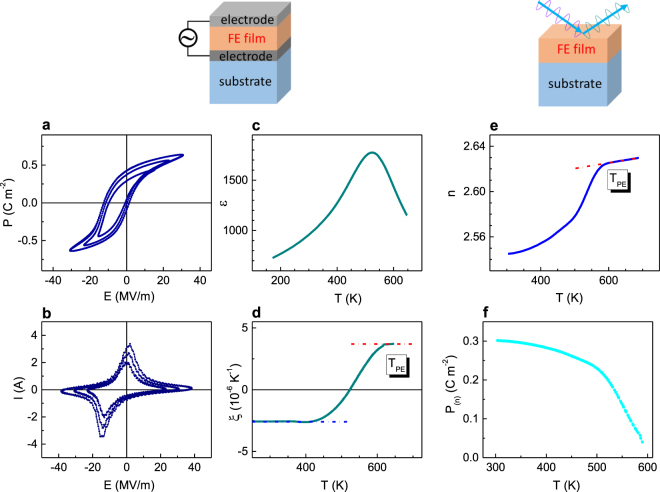
(**a**–**c**) Electrical properties measured in the Pt/PSTO/SRO/STO capacitor and (**e**,**f**) thermooptical behavior in the PSTO/STO film. (**a**,**b**) Dynamic (**a**) polarization – electric field and (**b**) current – electric field loops at frequency 1 kHz. (**c**) Dielectric permittivity as a function of temperature at frequency 10 kHz. (**d**) Derivative of inverse permittivity as a function of temperature. Dashed lines show fits to the Curie-Weiss law. (**e**) Refractive index *n* as a function of temperature measured at the photon energy 2 eV. Dashed line shows the high-temperature PE behavior. (**f**) Ferroelectric polarization extracted from the data in (**e**).

Epitaxial cube-on-cube-type PSTO films (thickness 100 nm) were grown by pulsed laser deposition directly on (001) SrTiO_3_ (STO) substrates or using epitaxial SRO bottom layer (see Methods and Supplementary section S1, Table S1, and Fig. S1). The films are pseudomorphic to the substrates, experience biaxial compressive in-plane strain, and exhibit out-of-plane elongation compared to bulk PSTO. The Pt/PSTO/SRO/STO capacitor is characterized electrically (Fig. 1a–d) and the electrodeless PSTO/STO film is studied by spectroscopic ellipsometry (see Methods, Supplementary section S2, and Fig. 1e,f). The room-temperature dynamic polarization – electric field (*P*-*E*) loops (Fig. 1a) and the corresponding current – electric field (*I*-*E*) loops (Fig. 1b) evidence the presence and switching of ferroelectric (FE) polarization in PSTO. The switchable polarization > 0.6 C·m^−2^ in the film exceeds spontaneous polarization in polycrystalline PSTO^37^, consistent with the effect of substrate-induced strain in ferroelectrics^38,39^. The real part of the dielectric permittivity, ε, extracted from the impedance measurements (see Methods and analysis in ref.^37^), possesses a broad peak in its temperature dependence (Fig. 1c). The peak indicates a phase transition. Because epitaxial strain and small thickness of ferroelectric films significantly affect dielectric peaks, we use derivative of inverse permittivity ξ = *d*(*ε*^−1^)/dT (Fig. 1d) to accurately identify the nature and temperature of transition^37^. The high-temperature Curie-Weiss behavior [ξ = *const* (*T*)] implies the paraelectric state (PE) and the low-temperature Curie-Weiss behavior is consistent with the FE state. The temperature, at which derivative deviates from the PE line on cooling, corresponds to the PE-FE transition temperature, *T*_PE_. The temperature *T*_PE_ ≈ 600 K in the strained epitaxial film is significantly higher than *T*_PE_ ≈ 430 K in polycrystalline PSTO films and ceramics, in agreement with ferroelectric strain-temperature diagrams^37,40^.

The PSTO/PTO film is transparent at photon energies < 3 eV (Supplementary Fig. S5). The film’s thermooptical behavior is investigated at the photon energy 2 eV, i.e., in the transparency range. A weak linear dependence *n*(*T*) at the temperatures > 600 K (Fig. 1e) indicates the PE state in the PSTO/STO film, similarly to that in ferroelectric crystals^25–36^. As shown previously, deviation from the linear dependence and a decrease of *n* are caused by appearance of polarization in perovskite oxide ferroelectrics and related materials^27–34^. Here, the temperature, at which refractive index deviates from the PE line on cooling, corresponds to the PE-FE transition. Remarkably, the temperature *T*_PE_, determined thermooptically in the PSTO/STO film (Fig. 1d), practically coincides with that determined electrically in the Pt/PSTO/SRO/STO capacitor (Fig. 1e).

Compared to the refractive index *n*_0_ in the high-temperature PE state (polarization *P* = 0), the index *n* is smaller in the FE state (*P* ≠ 0) due to strong quadratic electro-optic effect^41^. The relationship between the polarization magnitude *P*_(*n*)_ and refractive index is approximately^41^.

1 $$(\frac{1}{{n}^{2}}-\frac{1}{{n}_{0}^{2}})=g{P}_{(n)}^{2}$$

where *g* is the quadratic electro-optic coefficient, and the tensor nature of the coefficient *g* and Equation (1) is omitted for simplicity. The polarization magnitude *P*_(*n*)_ is estimated from the dependence *n*(*T*) using the expression (1), typical coefficient *g* ≈ 0.1 m^4^C^−2^, and *n*_0_ at *T*_PE_ (Fig. 1f). A good agreement between the room-temperature polarization *P*_(*n*)_ in PSTO/STO and spontaneous polarization in Pt/PSTO/SRO/STO further validates thermooptical analysis as a tool to detect ferroelectricity in electrodeless films.

### Ferroelectricity in ultrathin electrodeless films

**Figure 2:**
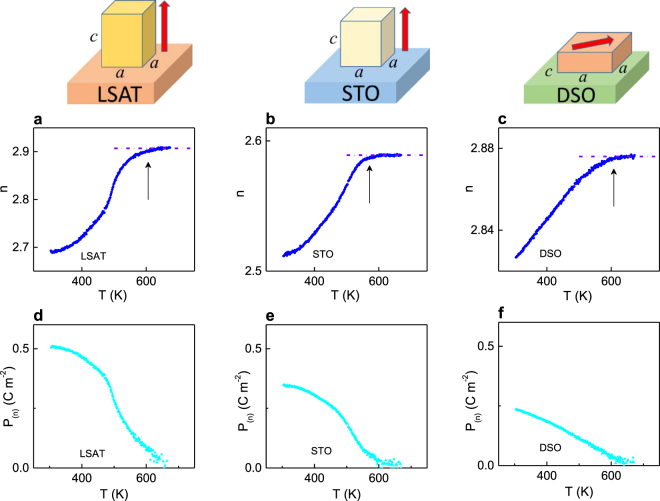
(**a**–**c**) Refractive index *n* as a function of temperature *T* measured at photon energy of 2 eV on heating in the PSTO films on (**a**) LSAT, (**b**) STO, and (**c**) DSO substrates. Dashed lines indicate the high-temperature paraelectric behavior. Arrows point to the ferroelectric transition. (**d**,**f**) Polarization *P*_(*n*)_ estimated from the refractive index in the PSTO films on (**d**) LSAT, (**e**) STO, and (**f**) DSO substrates. The unit cells of the films on STO, LSAT, and DSO and the polarization direction therein are schematically drawn on the top side.

PSTO films with thicknesses of ∼5 nm were deposited onto (001) (La_0.18_Sr_0.82_)(Al_0.59_Ta_0.41_)O_3_ (LSAT), (001) SrTiO_3_ (STO), and (011)DyScO_3_ (DSO) substrates. The pseudomorphic growth and cube-on-cube-type epitaxial relationships [100](001)PSTO|| [100](001)STO (or LSAT) and [100](001)PSTO|| [110](011) DSO are revealed for these films (Supplementary section S1, Figs S2 and S3). The in-plane lattice parameters *a* of the films are equal to those on the substrates surfaces. The in-plane compression, out-of-plane elongation (*a* < *c*), and enhanced tetragonality compared to the tetragonal bulk PSTO cell are obtained in the films on LSAT and STO. The crystal structure of the films suggests the out-of-plane direction of ferroelectric polarization. The in-plane tensile strain and out-of-plane shrinkage, resulting in the tetragonality being negative (*a* > *c*), are obtained on DSO. The polarization lies in the plane parallel to the substrate surface in PSTO/DSO. The unit cells and polarization are schematically illustrated in the upper part of Fig. 2.

With the absence of electrodes, the ferroelectric properties of the PSTO films were studied by analyzing their thermooptical behavior. The optical absorption spectra show that the films are transparent at the energies < 3 eV: the absorption coefficient is very small, only *∼*10^3^ cm^−1^, in this spectral region (Supplementary Fig. S6).

The refractive index *n* at 2 eV significantly increases with temperature in the PSTO films (Fig. 2a–c). The growth of *n* slows and a linear *n*(*T*) is found at the high temperatures *T* > (550–650) K, implying the PE state (as shown by dashed lines in Fig. 2a–c). This behavior of *n*(*T*) exactly matches observations in Fig. 1e and indicates the FE-PE phase transition and ferroelectricity in the films. The transition temperature *T*_PE_ in the 5-nm-thick film on STO is approximately the same as in the thicker PSTO/STO film (Fig. 1e). The temperature *T*_PE_ is higher in the film on LSAT compared to that on STO (Fig. 2a,b), in excellent agreement with the larger strain magnitude on LSAT.

The polarization *P*_(*n*)_ is estimated from the dependence *n*(*T*) using the expression (1) as described above (Fig. 2d–f). We note that, in contrast to electrical measurements of polarization, the quadratic nature of electro-optic effect (1) allows determining the in-plane and out-of-plane polarizations in the same way. Additionally to the estimations, the crystal structure of the films implies that polarization direction is normal to the substrate surface in the PSTO films on LSAT (Fig. 2d) and STO (Fig. 2e), and it is parallel to the substrate surface in the PSTO film on DSO (Fig. 2f). Again, the room-temperature polarization *P*_(*n*)_ in the 5-nm-thick film on STO (Fig. 2e) is approximately equal to that in the thicker PSTO/STO film (Fig. 1f). The polarization is larger in the film on LSAT compared to that on STO (Fig. 2d,e), conforming with the larger compressive strain on LSAT. Likewise, tensile in-plane strain explains relatively small polarization in the film on DSO (Fig. 2f).

The self-consistent results in Fig. 2 evidence ferroelectricity in the PSTO films despite the small film thickness and the absence of screening electrodes. The existence of the in-plane polarization in the electrodeless film on insulating DSO substrate is not surprising as it does not require screening. However, large out-of-plane polarization in the films on LSAT and STO implies efficient screening not only on the film surfaces, but also at the film-substrate interfaces. We suggest that free charge carriers inside the films may provide sufficient internal screening. Next, we theoretically analyze such mechanism.

### Internal screening

**Figure 3:**
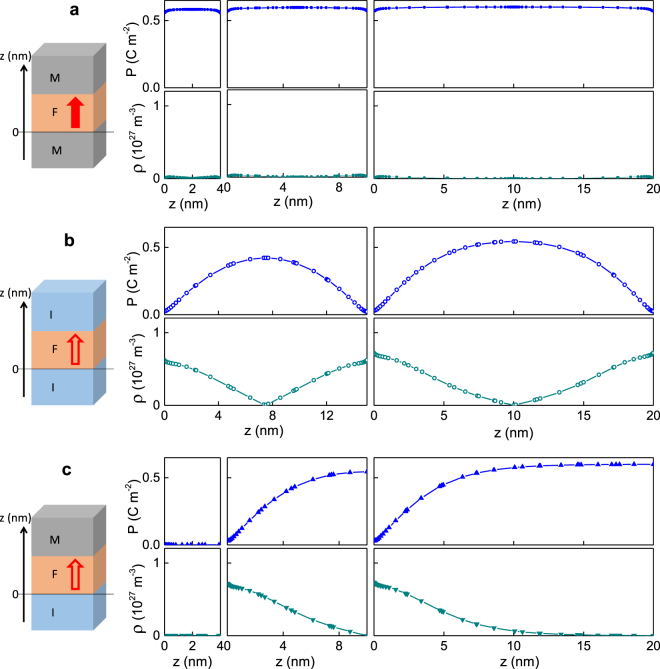
Polarization *P* and density *ρ* of screening charge carriers as a function of the out-of-plane coordinate *z* in the film sandwiched between (**a**) perfect electrodes (thickness is 4, 10, and 20 nm), (**b**) insulators (thickness is 15 and 20 nm), and (**c**) the bottom insulator and perfect top electrode (thickness is 4, 10, and 20 nm).

A model considered a single-domain ferroelectric film, which possesses out-of-plane (normal to substrate surface) polarization and contains free charge carriers^8,9,42,43^. The polarization *P*(*z*) was calculated using the Landau-Ginzburg-Devonshire theory with the Euler-Lagrange equation and three types of electrostatic boundary conditions: the film is short-circuited between perfect conducting electrodes (Fig. 3a); open-circuited and sandwiched between two insulators (Fig. 3b); and sandwiched between an insulator and conducting electrode (Fig. 3c). Here *z* is the out-of-plane coordinate across the film from its bottom to the top. The density *ρ* (z) of free charge carriers was calculated considering the ferroelectric as a semiconductor. The critical thickness for ferroelectricity was determined as that corresponding to a second order ferroelectric-to-paraelectric transition. The details of modeling and numerical calculations are given in Supplementary section S3. The calculated polarization and density of screening charge carriers are presented in Fig. 3. For convenience of comparison, the scales of corresponding ordinates are similar everywhere.

In the film between two perfect conducting electrodes (Fig. 3a), the room-temperature critical thickness for ferroelectricity is very small: <0.4 nm, which is comparable with one unit cell of typical perovskite oxide ferroelectric. The polarization is nearly uniform across the film’s thickness, and the density of screening carriers is insignificant. The free charges in electrodes are mainly responsible for screening, whilst internal screening inside the film has a minor effect on the polarization and carrier density in this case.

When both electrodes are absent and the film is sandwiched between two perfect insulators (Fig. 3b), the critical thickness is mainly determined by the density of charge carriers inside the film. For the two similar 100-nm-thick insulating layers, the critical thickness is at least 12 nm. For thicknesses larger than the critical one, the polarization and screening charge carriers are distributed along the coordinate *z*. The polarization is at its maximum in the center of the film, and it decreases to zero at the film-insulator interfaces. A high density of screening carriers is found in the regions of the films adjacent to the insulators. The screening charges have opposite signs at the top and bottom of the film. The carrier density exceeds the intrinsic equilibrium carrier density by at least an order of magnitude at the film-insulator boundary, and it decreases in the central part of the film. We note that the obtained distribution *ρ*(*z*) is in contrast to a model assumption in ref.^44^, where the free charges were *a priori* placed at the film’s surfaces only and not in the film’s interior.

In the stack insulator-film-electrode (Fig. 3c), the critical thickness is also mainly determined by the density of charge carriers. For a 100-nm-thick bottom insulator and perfect top electrode, the calculated critical thickness is approximately 6 nm. Note that a 4-nm-thick film is paraelectric. At the absence of polarization, the screening charge is also absent in this film. The polarization increases from zero at the bottom film-insulator boundary to a maximum at the top film-electrode boundary in the films of larger thicknesses. The screening charge carriers are accumulated in thin layers adjacent to insulators in these films. Such a few nanometer thick layers with high density of carriers resemble two-dimensional carrier gases in different materials systems^45^.

Our calculations show that internal screening can lead to stabilization of ferroelectricity in films as thin as 5–6 nm at the presence of one electrode. As known, species adsorbed on the film surfaces can ensure surface screening, thus acting as an electrode^11,12,43,46^. A combined effect of the internal screening by charge carriers and surface screening by adsorbed species can stabilize ferroelectricity in our films on LSAT and STO. To verify this scenario, we vary density of adsorbed species using slow adsorption kinetics^47^ and fast thermally activated desorption.

### Effect of surface

**Figure 4:**
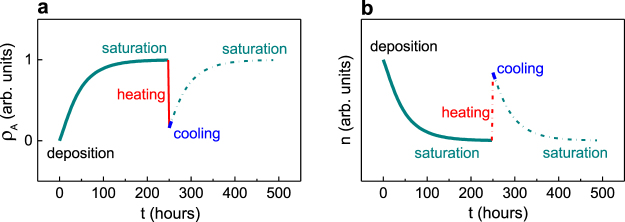
Schematics of the evolution of the (**a**) density of adsorbed species *ρ*_*A*_ and (**b**) refractive index *n* during the measurements.

We assume that the film surface is practically free of adsorbed species immediately after the high-temperature deposition and the density of adsorbed species *ρ*_*A*_ increases with time *t* (after film’s preparation) as [*ρ*_*A*_ = *ρ*_0_{1 + *A*exp(−*Bt*)}]^47^. For certain species, the coefficients *A* and *B* are functions of temperature and partial pressure of the species in a surrounding ambience. The kinetics of adsorption are qualitatively illustrated in Fig. 4a. The saturation of adsorption and thus the stabilization of polarization are achieved at the time *t*_*A*_ > 200 hours after film’s deposition. (The time *t*_*A*_ is found empirically here.) The density of adsorbed species decreases on heating via thermally activated desorption. For the heating rate of ∼3 K/min and maximum temperature of ∼(700–850) K used in our measurements, the heating duration Δ*t*_*H*_ ≈ 2.5 hours is short compared to the time *t*_*A*_. Therefore, the high-temperature desorption can be shown by a vertical line in Fig. 4a. Similarly, the duration Δ*t*_*C*_ of cooling is relatively short: Δ*t*_*C*_ ≈ 2.5 hours. Because of the slow adsorption kinetics, the density *ρ*_*A*_ increases insignificantly during the cooling run. The room-temperature density *ρ*_*A*_ is much smaller at the end of the cooling run than that at the beginning of the preceding heating run. A post-cooling recovery of the stabilized state occurs later.

The temporal evolution of the density of adsorbed species (Fig. 4a) determines changes in the boundary conditions and, hence, evolution of polarization and refractive index *n* (Fig. 4b). The boundary conditions correspond to those in the insulator-film-insulator stack (*ρ*_*A*_ = 0) immediately after deposition at *t* = 0. The film is paraelectric (large *n*) because its thickness (5 nm) is smaller than the critical one under these conditions. The conditions change to the insulator-film-electrode-type (large *ρ*_*A*_), the critical thickness decreases, and the film becomes ferroelectric (*n* drops) at *t* ≈ 250 hours. Upon heating, the intrinsic transition to the paraelectric state takes place (*n* increases). Concurrently, desorption results in the insulator-film-insulator-type conditions (small *ρ*_*A*_), which prevent the film from becoming polar with further relatively fast cooling (*n* remains large). The insulator-film-electrode-type (large *ρ*_*A*_) and ferroelectric state (small *n*) are recovered after waiting at room temperature to *t* = 500 hours.

Because adsorption saturates at the longer times than duration of measurements, the refractive index measured on heating (from the saturated state) is expected to be larger than that measured on consequent cooling (red and blue lines in Fig. 4b). Hence, a thermal hysteresis in *n*(*T*) should exist in the films on LSAT and STO, but not on DSO (no surface screening).

**Figure 5:**
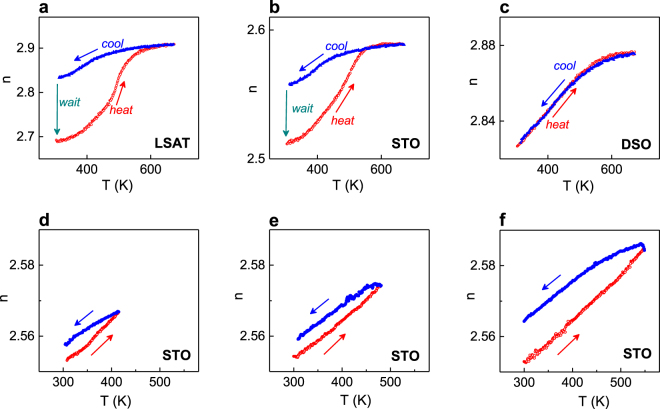
Refractive index *n* as a function of temperature *T* measured first on heating and on consequent cooling in the PSTO films on (**a**) LSAT, (**b**,**d**–**f**) STO, and (**c**) DSO substrates. Arrows show the directions of the temperature change. Dashed arrows in (**a**,**b**) show room-temperature recovery of *n* within several days after cooling. The heating temperatures are (**d**) 415K, (**e**) 480K, and (**f**) 550K.

Our experimental observations in Fig. 5 clearly support the role of surface. When the films on LSAT and STO are cooled from the high-temperature PE state, they exhibit refractive index *n*, which is significantly larger than that obtained in the preceding heating run (Fig. 5a,b). Remarkably, the refraction is recovered afterwards, within several days, when the films are kept at room temperature (marked as “wait” in Fig. 5). In contrast to the refraction hysteresis in the films on LSAT and STO, such a hysteresis is absent in the film on DSO (Fig. 5c). Additionally, as follows from the schematics in Fig. 4, the difference between the room-temperature values of *n* in thermal hysteresis should increase with increasing maximum heating temperature because of the corresponding larger fraction of desorbed species. To verify this expectation, thermal cycling of PSTO/STO was performed using approximately the same room-temperature stabilized refractive index as a starting point and by heating the film to different temperatures. The expected tendency to stronger hysteresis for higher heating temperatures is clearly confirmed in the measurements (Fig. 5d–f).

Finally, we note that at the presence of 180° domains in ultrathin ferroelectric PbTiO_3_ films, the phase transition temperature decreased by ∼200 K with decreasing film thickness from 100 to 5 nm and the polarization appeared on the real-time scale during cooling^17,18^. In contrast, the phase transition temperatures *T*_PE_ are similar, exceeding that in bulk, in our 100-nm-thick and 5-nm-thick PSTO films on STO, and the temperature *T*_PE_ is still higher in the 5-nm-thick PSTO/LSAT film. Additionally, the polarization stabilization occurs on the time scale of several days in our films. The high phase transition temperatures and very slow build-up of polarization imply the absence of 180° domains here.

## Conclusions

Our modeling and experimental results strongly suggest that a single-domain ferroelectric state can be stabilized in ultrathin films by redistribution of the charge carriers therein. This mechanism is favored in asymmetric stacks, where the film is sandwiched between the screening electrode or surface layer and perfect insulator. The experiments performed here validate this mechanism in the films of PSTO. We note that other ultrathin ferroelectric films (of KNbO_3_, NaNbO_3_, KTaO_3_, BaTiO_3_, and PbTiO_3_) also exhibit similar behavior, highlighting its universal character^48–52^.

For uncoated ultrathin films grown onto insulating substrates, the internal screening is highly sensitive to the state of the film surface and to the density of charge carriers in the films. This behavior can enable such applications as chemical sensors, radiation detectors, and others. For metal-coated films, the internal screening is more robust, and it ensures strongly confined high-density charges, whose properties may resemble those of two-dimensional carrier gases. We believe that internal screening has high potential for applications and deserves further investigations.

## Methods

Epitaxial films were grown by pulsed laser deposition using an excimer laser (wavelength 248 nm). Custom-made (Institute of Solid State Physics, Riga, Latvia) ceramics were used as targets. The energy density of the laser radiation incident on the target was 2.0 J/cm^2^. The target-to-substrate distance of 35 mm, substrate temperature of 973 K, and ambient oxygen pressure of 20 Pa were used during the growth. The oxygen pressure was raised to 800–1000 Pa during post-deposition cooling, realized at a rate of 5 K/min. The films were deposited onto epitaxially polished single-crystal substrates, purchased from the MTI Corporation.

The crystal structure of the films was analyzed by high-resolution x-ray diffraction on a Bruker D8 diffractometer using Cu-Kα radiation. The crystal phases, perfection, orientation, epitaxy, and lattice parameters of the films were studied by inspection of the film diffractions and use of the substrate diffractions as a reference. The lattice parameters were determined using LEPTOS software. The details of crystal structure were inspected using electron diffraction on a JEOL 2200FS TEM. The cross-sectional samples were prepared by focused ion beam milling.

The electrical characterization was performed using a TF 2000E Analyzer (aixACCT Systems GmbH), an HP 4268 A Precision LCR Meter, and a Linkam TSE350 MultiProbe stage.

The optical properties of the films were characterized by variable-angle spectroscopic ellipsometry using J. A. Woollam ellipsometers. The ellipsometric data were collected at room temperature in a vacuum or dry nitrogen over a spectral range from 0.74 to 9.0 eV. High-temperature measurements were carried out in the air over a spectral range from 0.74 to 5.0 eV. Data analysis was performed using the WVASE32 software package and a procedure developed by J. A. Woollam Inc.

The datasets generated and/or analyzed during the current study are available from the corresponding author on reasonable request.

## Electronic supplementary material

Supplementary Information
