# Supplementary material for: Thermooptical evidence of carrier-stabilized ferroelectricity in ultrathin electrodeless films
Source: Sci Rep. 2018 May 31;8:8497. doi: 10.1038/s41598-018-26933-0 (PMC5981214; doi:10.1038/s41598-018-26933-0)
Supplement: Supplementary file 1 — Supplementary Information [file 41598_2018_26933_MOESM1_ESM.pdf]

Supplementary Information

to

Thermooptical evidence of carrier-stabilized ferroelectricity in ultrathin electrodeless films

O. Pacherova[1], D. Chvostova[1], T. Kocourek[1], M. Jelinek[1], A. Dejneka[1], E. Eliseev[2], A. Morozovska[3], M. Tyunina[4,1] *

[1]*Institute of Physics of the Czech Academy of Sciences, Na Slovance 2, 18221 Prague, Czech Republic*
[2]*Institute of Problems for Material Sciences, NAS of Ukraine, 03028 Kyiv, Ukraine*
[3]*Institute of Physics, NAS of Ukraine, 03028 Kyiv, Ukraine*
[4]*Microelectronics Research Unit, Faculty of Information Technology and Electrical Engineering, University of Oulu, P. O. Box 4500, FI-90014 Oulu, Finland*
* E-mail: marina.tjunina@oulu.fi; tjunina@fzu.cz

S1. Crystal structure

PSTO ceramics possess the room-temperature tetragonal crystal structure and lattice parameters $a = b \approx 0.392$ nm and $c \approx 0.396$ nm [1]. Compared to a cubic bulk PSTO cell (lattice parameter $a_0 = (a^2c)^{1/3} \approx 0.393$ nm), a biaxial in-plane misfit strain [$s_a = a_s/a_0$ -1] is expected in a cube-on-cube-type epitaxial film of PSTO, coherently grown onto a cubic substrate with the lattice parameter a_s. The film-substrate mismatch between coefficients of thermal expansion can lead to an additional strain arising on cooling from the high temperature of deposition to room temperature. Here, this thermal strain is omitted for simplicity. The in-plane misfit strain s_a, in-plane and out-of-plane lattice parameters a and c, respectively, tetragonality (c/a-1), and unit-cell volume $V = a^2c$ are estimated for the films coherent to the square surface cells of LSAT(001), STO(001), and DSO(011) substrates (Table S1).

Table S1. Theoretical in-plane misfit strain s_a, lattice parameters a and c, tetragonality (c/a-1), and unit cell volume in epitaxial PSTO films on different substrates compared to those in bulk PSTO.

	substrate	s_a, %	a, nm	c, nm	(c/a-1), %	V, 10^{-3} nm^3
bulk			0.392	0.396	1.0	60.8
film	LSAT	-1.6	0.387	0.399	3.3	59.8
film	STO	-0.7	0.391	0.396	1.4	60.4
film	DSO	+0.3	0.394	0.392	-0.6	61.0

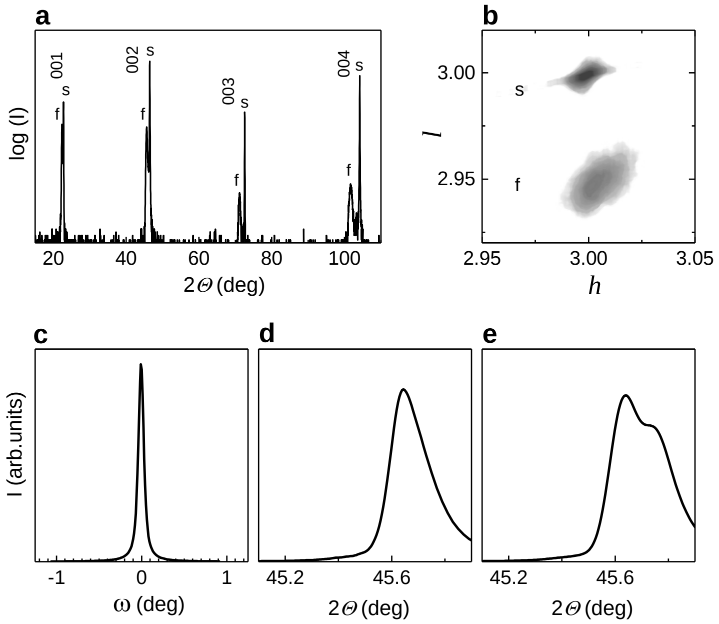

Figure S1. (a, d, e) Θ-2Θ x-ray diffraction scans, (b) reciprocal space map, and (c) rocking curve around the (004) perovskite diffraction in the 100-nm-thick PSTO films on (a-d) bare and (e) SRO-coated STO. Diffractions from the film and substrate are marked by "f" and "s" in (a, b).

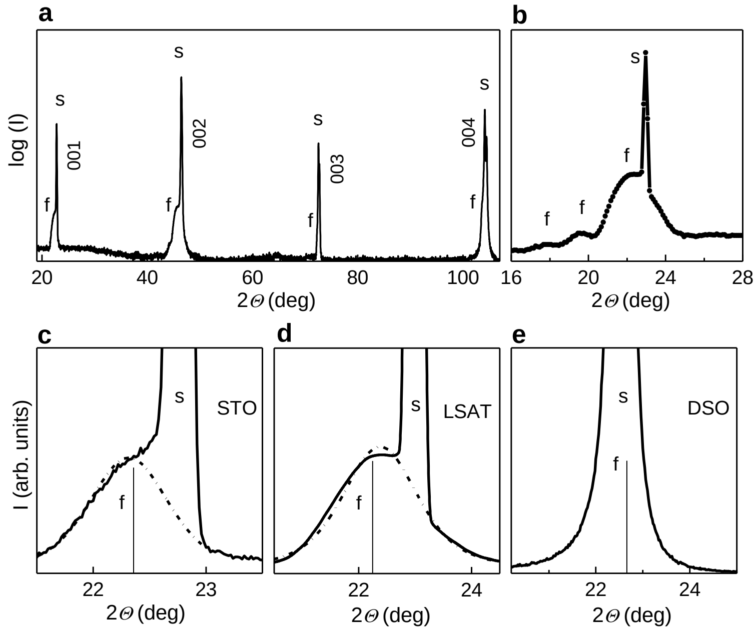

Figure S2. Θ-2Θ x-ray diffraction patterns in the PSTO films on (a, c) STO, (b, d) LSAT, and (e) DSO substrates. Diffractions from the films and substrates are marked by f and s, correspondingly. The panels (b-e) show scans around perovskite (001) diffractions. The dashed lines in (c-e) indicate diffractions from the films. The theoretical positions of the peaks are shown by straight vertical lines.

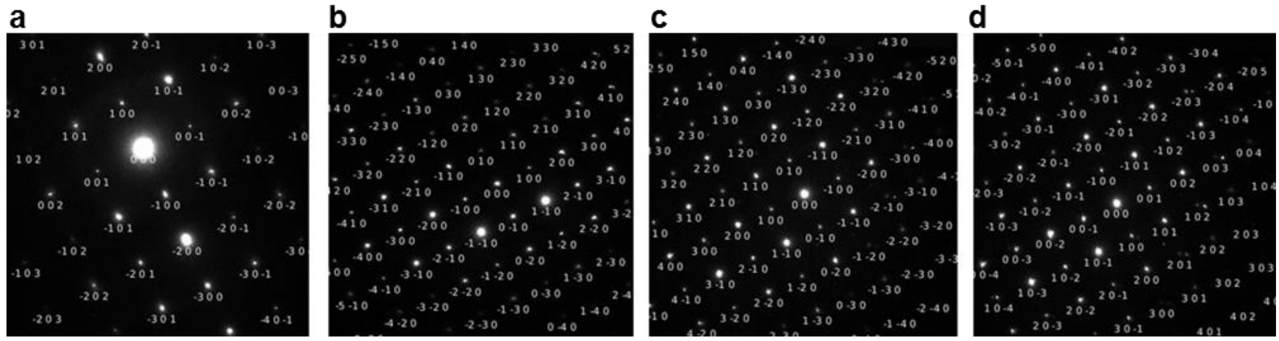

Figure S3. The (a) selected area electron diffraction and (b-d) nanobeam electron diffraction patterns from the films on (a-b) STO, (c) LSAT, and (d) DSO. The *hkl* indices are shown close to the corresponding spots.

The grown films are cube-on-cube-type epitaxial, with the in-plane lattice parameters equal to those on the substrates surfaces. The out-of-plane lattice parameters of the films on STO are similar, indicating the same in-plane compressive strain in these films. The larger in-plane compression and the in-plane tension are detected in the films on LSAT and DSO, correspondingly. The strains in the films are close to the theoretical estimations (Table S1).

S2. Spectroscopic ellipsometry.

The optical properties of the thin films were probed using variable-angle spectroscopic ellipsometry (VASE). This is a rapidly advancing method experiencing remarkable progress in both the equipment and the methodological aspects. The method is superior in terms of accuracy compared to reflection and/or transmission analysis. State-of-the-art VASE allows for high-precision studies of optical properties of films with a thickness of only a few atomic layers and of separate macromolecules on top of arbitrary substrates [2]. Accurate analyses of ellipsometric data for wide bandgap materials are only possible if the spectral range is sufficiently expanded into the high-energy (UV) region. For this reason, an J. A. Woollam ellipsometer with an extended photon energy range is the most suitable choice.

Ellipsometric data were collected using a variable-angle rotating-analyzer spectroscopic ellipsometer over a spectral range from 0.74 eV to 9.0 eV. Each sample was measured at two angles of incidence ($\Theta = 65^o, 70^o$). This allowed us to obtain spectra of ellipsometric angles (Δ, ψ) with an excellent accuracy of 0.2 deg for Δ and 0.04 deg for ψ [3]. VASE data analysis was based on numerical inversion and minimization of possible artifacts. The analysis was performed using the WVASE32 software package [4]. The complex refractive index as a function of photon energy (here - optical properties for brevity), the thickness of the films, and the surface roughness were obtained from the analysis.

For data processing, the experimental ellipsometric spectra were fitted using a model considering a stack of a semi-infinite substrate, a film, a surface roughness layer, and ambient air. The parameterization of the initial dielectric functions of the films was based on the multi-oscillator model. The optical properties of the surface roughness layer were represented by a Bruggeman effective medium approximation [5]. The initial optical spectra and thickness of the film and surface roughness layer were extracted using a least-square regression analysis. After a refinement of the initial optical properties and layer thickness, the thickness was fixed and numerical inversion was used to extract optical properties from the measured spectra of ellipsometric angles Δ and ψ. The dielectric functions and the optical properties of the substrates were determined from separate measurements. The data analysis accounted for the surface roughness layer. Several substrates of each type from MTI Corp. were studied. The optical properties of the substrates were used in the VASE data processing.

The mean square error (MSE) for thin-film samples was in the range of 0.1 – 0.25. This excellent accuracy indicates a high reliability of the obtained results [6]. A typical example of a VASE data fits is shown in Fig. S4.

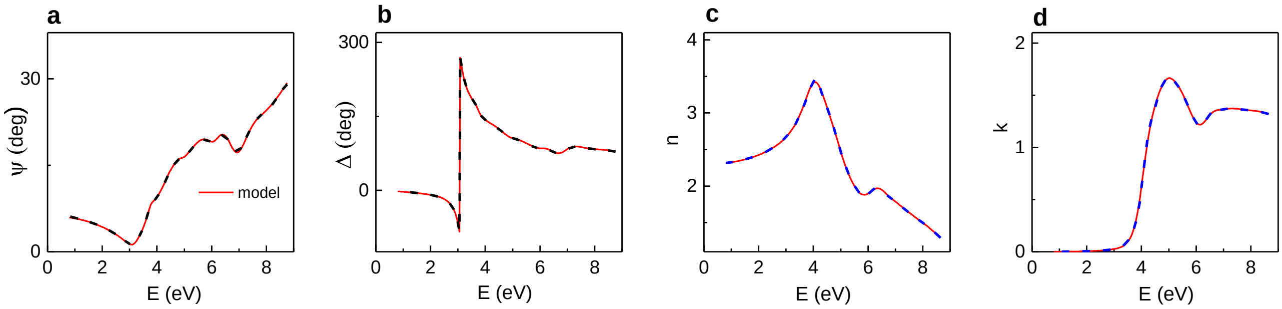

Figure S4. Ellipsometric angles (a) ψ and (b) Δ as a function of photon energy. Dashed black curves and solid red curves show experimental data and fits, correspondingly. (c) Index of refraction *n* and (d) extinction coefficient *k* as a function of photon energy determined using oscillator model (dashed curves) and point-by-point numerical inversion analysis (dashed blue curves).

The absorption coefficient α was obtained using the relationship $\alpha = 4\pi k/\lambda$, where k is the extinction coefficient (the imaginary part of the complex index of refraction) and λ is the wavelength of the light.

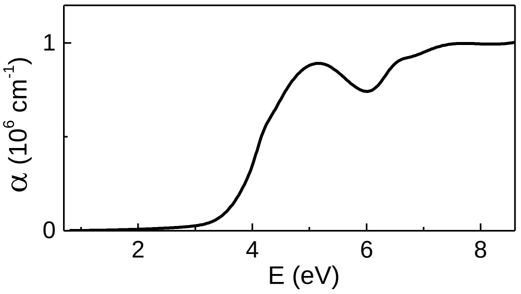

Figure S5. Absorption coefficient α as a function of photon energy E in the 100-nm-thick PSTO film on STO.

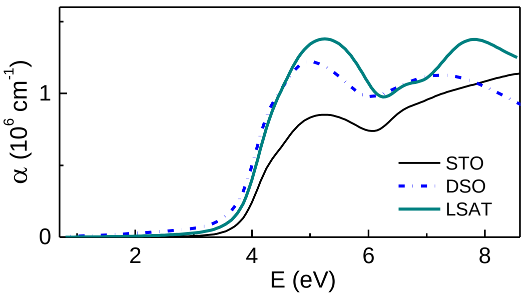

Figure S6. Absorption coefficient α as a function of photon energy E in the 5-nm-thick PSTO films on STO, LSAT, and DSO substrates.

S3. Modeling

A single-domain ferroelectric film with the out-of-plane (normal to substrate surface) polarization $P_3(z)$ was considered. Here z is the out-of-plane coordinate across the film from its bottom to the top. The polarization was calculated using the Landau-Ginzburg-Devonshire (LGD) theory with the Euler-Lagrange equation and boundary conditions given as follows:

$$\begin{cases} \alpha P_3 + \beta P_3^3 + \gamma P_3^5 - g\dfrac{\partial^2 P_3}{\partial z^2} = -\dfrac{\partial \varphi}{\partial z}, \\ \left.\left(P_3 + \lambda\dfrac{\partial P_3}{\partial z}\right)\right|_{z=0} = 0, \quad \left.\left(P_3 - \lambda\dfrac{\partial P_3}{\partial z}\right)\right|_{z=L} = 0. \end{cases} \quad (1)$$

Here, $\alpha = \alpha_T(T - T_c)$, β, and γ are the expansion coefficients of the LGD potential, $g = g_{33}$ is the tensor component of gradient energy coefficients, λ is the extrapolation length [7], and L is the thickness of the ferroelectric film. The electric potential φ was found from the Poisson equation:

$$\varepsilon_{33}^b \frac{\partial^2 \varphi}{\partial z^2} = \frac{1}{\varepsilon_0}\left(\frac{\partial P_3}{\partial z} - \rho(\varphi)\right), \quad (2)$$

where ε_{33}^b is the background permittivity of the ferroelectric material [8] and $\rho(\varphi)$ is the density of space charge in the ferroelectric film. The potential φ satisfies the 1D Laplace equation in the dielectric environment with relative permittivity ε_{33}^a:

$$\varepsilon_{33}^a \frac{\partial^2 \varphi}{\partial z^2} = 0\,. \quad (3)$$

The calculations were performed for three types of electrostatic boundary conditions: the film is (a) short-circuited between perfect conducting electrodes; (b) open-circuited and sandwiched between two insulators; and (c) sandwiched between an insulator and conducting electrode. The electric potential is zero at the short-circuited interfaces, i.e.,

$$\varphi|_0 = \varphi|_L = 0 \quad (4)$$

for the film with two electrodes,

$$\varphi|_L = 0 \quad (5)$$

for the film with one top electrode, and

$$\varphi|_{-0} = \varphi|_{+0}, \quad \left.\left(-\varepsilon_0\varepsilon_a\frac{\partial\varphi}{\partial z}\right)\right|_{-0} = \left.\left(-\varepsilon_0\varepsilon_{33}^b\frac{\partial\varphi}{\partial z} + P_3\right)\right|_{+0} \quad (6)$$

for the film-insulator boundary. The conditions (6) assume clean non-conducting film-insulator interfaces, where free surface charge is absent and the electrical displacement is continuous [9-13]. The electric field outside the ferroelectric film was set to zero to prevent the energy of the system reaching infinity.

The density of free charge carriers in the ferroelectric film was calculated considering the ferroelectric as a donor-doped semiconductor with a thin donor level, whose activation energy is E_d [14-17]. The donor density is [18]:

$$N_d^+(\varphi) = N_{d0}\left(1 - f\left(E_d - E_F - q\varphi\right)\right), \tag{7}$$

where N_{d0} is the density of donor centers, $f(x) = \left(1 + \exp\left(x/k_B T\right)\right)^{-1}$ is the Fermi-Dirac distribution function, k_B = 1.3807×10^{-23} J/K, T is the absolute temperature, and E_F is the Fermi energy. The conduction band (CB) electron density is [14, 19]:

$$n(\varphi) = \int_0^\infty dE \cdot G_n(E) f\left(\varepsilon + E_C - E_F - q\varphi\right), \tag{8}$$

where E_C is the bottom of the CB. For an effective-mass density of states $G_n(E) \approx \frac{\sqrt{2m_n^3 E}}{2\pi^2\hbar^3}$, it takes the form

$$n(\varphi) \approx \left(\frac{m_n k_B T}{\hbar^2}\right)^{3/2} \frac{1}{\pi^2\sqrt{2}} \frac{\sqrt{\pi}}{2}\left(-\mathrm{Li}_{3/2}\left(-\exp\left(\frac{q\varphi + E_F - E_C}{k_B T}\right)\right)\right), \tag{9}$$

where $\mathrm{Li}_n(z) = \sum_{k=1}^{\infty} \frac{z^k}{k^n}$ is a polylogarithmic function. The Fermi level is found from the condition of electro-neutrality:

$$\rho(0) = N_{d0}^+ - n_0 = 0, \tag{10}$$

where $n_0 = \int_0^\infty dE \cdot G_n(E) f\left(E + E_C - F_F\right)$ and $N_{d0}^+ = N_{d0} f\left(E_F - E_d\right)$.

The critical thickness for ferroelectricity was considered as that corresponding to a second order ferroelectric-to-paraelectric transition. Because the spontaneous polarization and depolarizing field are very small in the immediate vicinity of the transition, a Debye approximation for the charge density can be used. For self-screening by free carriers with $\left|e\varphi/k_B T\right| << 1$, the Poisson equation acquires the form:

$$\frac{\partial^2 \varphi}{\partial z^2} - \frac{\varphi}{R_d^2} = \frac{1}{\varepsilon_0 \varepsilon_{33}^b} \frac{\partial P_3}{\partial z}, \tag{11}$$

where $R_d = \sqrt{\varepsilon_0 \varepsilon_{33}^b k_B T / \left(2e^2 n_0\right)}$ is the screening radius. The critical thickness was calculated for the above described three types of electrostatic boundary conditions.

Numerical simulations of the polarization and screening charge density were performed using the following parameters: α_T = 0.753×10^6 m/(F K), T_C = 700 K, β = 1.12×10^9 J m^5/C^4, γ = 10^9 J m^9/C^6, Q_{12} = -0.02 m^4/C^2, s_{11} = 7.0×10^{-12} Pa^{-1}, s_{12} = -2.0×10^{-12} Pa^{-1}, g_{33} = 5.0×10^{-10} m^3/F, ε_{33}^b = 10, ε_{33}^a = 300, and the intrinsic density of charge carriers in the film n_0 = 10^{25} m^{-3}. The extrapolation length λ is related to the properties of interfaces. The length is λ = 0 for the polarization P_3 = 0 at the interface, and it tends to infinity ($\lambda \to \infty$) for the boundary condition $\frac{\partial P_3}{\partial z} = 0$. In the absence of accurate

physical model for the length λ, rather small lengths are assumed for the film-insulator interfaces and larger lengths are assumed for the film-electrode interfaces, as specified below.

The three types of stacks were analyzed: electrode-film-electrode, insulator-film-insulator, and insulator-film-electrode. The critical thickness, polarization, and density of screening charge were calculated.

The critical thickness $L_{cr}^{(a)}$ for ferroelectricity in the film sandwiched between two perfect conducting electrodes is equal to

$$L_{cr}^{(a)} = \frac{g}{a}\left(\frac{1}{\lambda_1+\xi}+\frac{1}{\lambda_2+\xi}\right), \tag{12}$$

where $a=\alpha_T(T_C-T)+2q_{12}u_m$ is the coefficient α renormalized by misfit strain u_m (if any) and $\xi=\sqrt{\varepsilon_0\varepsilon_{33}^b g}$ is the correlation length [12]. This critical thickness exists for $a>0$, and it depends on temperature. Often, the correlation length ξ is small; therefore, $\lambda_m+\xi\approx\lambda_m$, and the expression (12) is approximated by

$$L_{cr}^{(a)} \approx \frac{g}{a}\left(\frac{1}{\lambda_1}+\frac{1}{\lambda_2}\right). \tag{13}$$

As shown in the expressions (12) and (13), the low-temperature ($T < T_C$) critical thickness is sensitive to the properties of the film-electrode interfaces, described by the extrapolation lengths λ_1 and λ_2. For the selected parameters and similar film-electrode interfaces ($\lambda_1 = \lambda_2 = 50$ nm), the calculated room-temperature critical thickness is very small: $L_{cr}^{(a)} \le 0.4$ nm. We note that the critical thickness can be additionally affected by such phenomena as interfacial band bending, formation of the Schottky barrier, and interface capacitance, which are ignored here for simplicity.

When both electrodes are absent and the film is sandwiched between two perfect insulators, the critical thickness $L_{cr}^{(b)}$ is equal to

$$L_{cr}^{(b)} \approx 2\kappa\arctan\left(\frac{\kappa}{\xi}\left(1+\mu\frac{2\xi+\lambda_1+\lambda_2}{\lambda_1+\lambda_2}\right)\right). \tag{14}$$

Here $\kappa\approx\sqrt{k_BT/\left(2e^2na\right)}$ is the effective screening length and $\mu=\frac{g}{a}\left(\frac{1}{\kappa^2}+\frac{1}{\xi^2}\right)$ is the dimensionless coefficient [13]. Typically, the effective screening length is much larger than the correlation length: $\kappa >> \xi$. The critical thickness is then approximately

$$L_{cr}^{(b)} \approx \pi\kappa\,. \tag{15}$$

For the selected parameters and the two similar 100-nm-thick insulating layers, the room-temperature single-domain ferroelectric state is found to be stable if the thickness of the film is at least 12 nm.

The calculations do not account for the materials' bandgaps. Qualitatively, considering the bandgaps of ≥ 3 eV in ferroelectrics and those of > 3 eV in most insulating substrates, the interfacial band alignment is expected to either have no effect or to prevent charge from leaking out of the film.

Because the substrates are usually low-permittivity dielectrics, the assumed absence of an electric field and, consequently, polarization outside the films is justified.

The stack insulator-film-electrode represents a case with one short-circuited interface and another open-circuited interface, where the boundary conditions (5) and (6) are valid. An analytical expression for the critical thickness takes the form (16):

$$L_{cr}^{(c)} \approx \kappa \arctan\left(\frac{\kappa}{\xi}\left(1+\mu\frac{2\xi+\lambda_1+\lambda_2}{\lambda_1+\lambda_2}\right)\right). \quad (16)$$

For the screening length larger than the correlation length ($\kappa >> \xi$), expression (16) is reduced to

$$L_{cr}^{(c)} \approx \pi\kappa/2 \equiv \sqrt{\pi^2 k_B T/\left(8e^2 na\right)}. \quad (17)$$

For the selected parameters, for a 100-nm-thick bottom insulator and perfect top electrode, the calculated critical thickness is approximately 6 nm.

References

[1] M. Tyunina, M. Plekh, M. Antonova, and A. Kalvane. *Ferroelectric transitions in epitaxial $Pb_{0.5}Sr_{0.5}TiO_3$ films studied by dielectric analysis.* Phys. Rev. B **84**, 224105 (2011).

[2] J. Hilfiker, C. Bungay, R. Synowicki, T. Tiwald, C. Herzinger, B. Johs, G. Pribil, J. A. Woollam. *Progress in spectroscopic ellipsometry: Applications from vacuum ultraviolet to infrared.* J. Vac. Sci. Technol. A **21**, 1103 (2003).

[3] B. Johs, C. M. Herzinge. *Quantifying the Accuracy of Ellipsometer Systems*. Phys. Stat. Sol. (c) **5**, 1301 (2008).

[4] *Guide to Using WVASE 32: Spectroscopic Ellipsometry Data Acquisition and Analysis Software* (J. A. Woollam Inc., 2008).

[5] H. Fujiwara, J. Koh, P. I. Rovira, R. W. Collins. *Assessment of effective-medium theories in the analysis of nucleation and microscopic surface roughness evolution for semiconductor thin films.* Phys. Rev. B **61**, 10832 (2000).

[6] J. Hilfiker, N. Singh, T. Tiwald, D. Convey, S. M. Smith, J. H. Baker, H. G. Tompkins. *Survey of methods to characterize thin absorbing films with Spectroscopic Ellipsometry.* Thin Solid Films **516**, 7979 (2008).

[7] C.-L. Jia, V. Nagarajan, J.-Q. He, L. Houben, T. Zhao, R. Ramesh, K. Urban, R. Waser. *Unit-cell scale mapping of ferroelectricity and tetragonality in epitaxial ultrathin ferroelectric films*. Nat. Mater. **6**, 64 (2007).

[8] A. K. Tagantsev, G. Gerra, and N. Setter. *Short-range and long-range contributions to the size effect in metal-ferroelectric-metal heterostructures*. Phys. Rev. B 77, 174111 (2008).

[9] Y. L. Li, S. Y. Hu, Z. K. Liu, and L. Q. Chen. *Effect of electrical boundary conditions on ferroelectric domain structures in thin films.* Appl. Phys. Lett. **81**, 427-429 (2002).

[10] I. I. Ivanchik. *On the microscopic theory of ferroelectrics.* Fiz. Tverd. Tela **3**, 3731 (1961).

[11] S. V. Kalinin, E. Karapetian, and M. Kachanov. *Nanoelectromechanics of piezoresponse force microscopy.* Phys. Rev. B **70**, 184101 (2004).

[12] E. A. Eliseev, A. N. Morozovska. *General approach to the description of the size effect in ferroelectric nanosystems.* J. Mat. Sci. **44**, 5149 (2009).

[13] E. A. Eliseev, S. V. Kalinin, A. N. Morozovska. *Finite size effects in ferroelectric-semiconductor thin films under open-circuited electric boundary conditions*. J. Appl. Phys. **117**, 034102 (2015).

[14] E. A. Eliseev, A. N. Morozovska, G. S. Svechnikov, V. Gopalan, and V. Ya. Shur. *Static conductivity of charged domain walls in uniaxial ferroelectric semiconductors*. Phys. Rev. B **83**, 235313 (2011).
[15] M. Y. Gureev, A. K. Tagantsev, and N. Setter. *Head-to-head and tail-to-tail 180° domain walls in an isolated ferroelectric.* Phys. Rev. B **83**, 184104 (2011).
[16] E. A. Eliseev, A. N. Morozovska, G. S. Svechnikov, P. Maksymovych, S.V. Kalinin. *Domain wall conduction in multiaxial ferroelectrics: impact of the wall tilt, curvature, flexoelectric coupling, electrostriction, proximity and finite size effects.* Phys. Rev. B **85**, 045312 (2012).
[17] S. M. Sze, *Physics of Semiconductor Devices* (Wiley-Interscience, New York, 1981).
[18] N. W. Ashcroft, N. D. Mermin, *Solid state physics* (Holt, Rinehart and Winston, New York, 1976).
[19] A. I. Anselm, *Introduction to semiconductor theory* (Mir, Moscow, Prentice-Hall, Englewood Cliffs, NJ, 1981).
